# Insights Gained From the Study of Pediatric Systemic Lupus Erythematosus

**DOI:** 10.3389/fimmu.2018.01278

**Published:** 2018-06-05

**Authors:** Mindy S. Lo

**Affiliations:** ^1^Division of Immunology, Boston Children’s Hospital, Boston, MA, United States; ^2^Department of Pediatrics, Harvard Medical School, Boston, MA, United States

**Keywords:** systemic lupus erythematosus, pediatric lupus, monogenic lupus, complement deficiency, DNASE1L3, TREX1, interferonopathy, rasopathy

## Abstract

The pathophysiology of systemic lupus erythematosus (SLE) has been intensely studied but remains incompletely defined. Currently, multiple mechanisms are known to contribute to the development of SLE. These include inadequate clearance of apoptotic debris, aberrant presentation of self nucleic antigens, loss of tolerance, and inappropriate activation of T and B cells. Genetic, hormonal, and environmental influences are also known to play a role. The study of lupus in children, in whom there is presumed to be greater genetic influence, has led to new understandings that are applicable to SLE pathophysiology as a whole. In particular, characterization of inherited disorders associated with excessive type I interferon production has elucidated specific mechanisms by which interferon is induced in SLE. In this review, we discuss several monogenic forms of lupus presenting in childhood and also review recent insights gained from cytokine and autoantibody profiling of pediatric SLE.

## Introduction

Systemic lupus erythematosus (SLE) is typically thought of as an autoimmune disease that affects women of childbearing age. However, 10–20% of patients have onset of disease in adolescence or younger. Referred to variably as childhood-onset or pediatric SLE (pSLE), these patients represent a subset with distinct characteristics. Clinically, children with pSLE typically have more severe disease and organ damage. From a pathophysiologic perspective, early onset of disease may also hint at a stronger genetic contribution. Over the years, identification of rare gene variants causing lupus-like phenotypes, so-called monogenic lupus, have in turn offered insights into lupus pathogenesis as a whole.

This review will summarize recent insights into the genetic origins of SLE that have been demonstrated by the study of pSLE patients. Recent work on molecular profiling and biomarker development in pSLE will also be reviewed here.

## Clinical Aspects of pSLE

There are limited data on precise incidence and prevalence of SLE in children, in part, because age definitions for “childhood-onset” vary. One U.S. study estimated a prevalence of 9.73 per 100,000 children, with an incidence rate of 2.22 cases/100,000/year (1). Non-White children show higher prevalence of disease (1). Non-Caucasian children also have higher rates of renal involvement and younger age of onset (2). African-American and Hispanic children with pSLE have higher rates of end-stage renal disease and death according to a survey of U.S. hospital admissions data (3). A large Canadian cohort study of pSLE patients followed over time also found that Afro-Caribbean children had higher early disease damage and a higher trajectory of damage accrual (4). These results are largely similar to demographic associations described in adults with SLE (5, 6).

In contrast to the similarities in racial and ethnic patterns, female sex predominance is less significant in children as compared to adults. SLE has been found in multiple studies to disproportionately affect women at a ratio of ~9 to 1, especially among patients of peak child-bearing age (7). This pattern is strong evidence for the importance of a hormonal role in the pathogenesis of SLE. In children, estimated female:male ratios range from 3.6–5.3 to 1 (1, 8, 9). The sex predominance becomes less and less pronounced with younger age of onset, and children with prepubertal development of SLE show essentially no sex bias (10). With the hormonal influence presumably removed, pSLE patients represent a unique opportunity to study the genetic contributions to lupus pathogenesis.

## Monogenic Lupus

### Complement

The classic example of single gene mutation leading to a lupus-like phenotype (so-called “monogenic lupus”) is that of complement deficiency. Hypocomplementemia was recognized as a common laboratory abnormality of SLE relatively early on, thought to be related to consumption and/or tissue deposition. Subsequently, however, the first familial cases of SLE in children due to C1 deficiency were described in the 1970s (11). Lupus-like presentations have now been associated with inherited deficiencies in many classical pathway complement components, including C1q, C1r, C1s, C2, C3, C4A, and C4B (12–15). Characteristically, lupus in these patients develops at an early age and many have severe cutaneous involvement (16). Extrapolating from these observations, it has also been noted that SLE patients as a whole are more likely to have lower copy numbers of *C4A* and *C4B* genes as compared to healthy populations, and this is especially striking in earlier onset disease (17, 18).

In the absence of normal complement regulation, inadequate clearance of apoptotic debris may encourage presentation of self-antigen. Aberrant apoptosis and clearance is now thought to be an important mechanism in lupus pathogenesis. Complement proteins facilitate the appropriate clearance of immune complexes that can lead to tissue damage in SLE and may also regulate the production of inflammatory cytokines by immune cells (16). These hypotheses, and the clinical presentations of complement deficiency, are reviewed in detail elsewhere (16, 19, 20).

Circulating autoantibodies against complement proteins such as C1q and C3b can be found deposited in the kidneys of lupus nephritis patients, provoking inflammation and mediating tissue damage (21, 22). The titer of anti-C1q antibodies correlates with disease activity in children with lupus nephritis (23). However, it is not clear if the depletion of C1q by these autoantibodies also contributes to immunopathogenesis of SLE.

The use of fresh frozen plasma (FFP) to replete complement components may be effective for patients with inherited complement deficiency (24, 25). One recent case series describes three children with C1q deficiency and severe SLE. In all three patients, treatment with FFP allowed rapid recovery and the ability to discontinue steroids (26). Whether repletion of complement is useful for patients without inherited deficiency remains to be seen. In a recent intriguing report, an adolescent girl with SLE and severe hypocomplementemia but no identified genetic deficiency was noted to have effective but transient responses to B cell depletion with rituximab (27). The authors then administered FFP together with ofatumumab to facilitate complement-mediated B cell lysis, resulting in more profound and longer lasting B cell depletion. Her complement levels later recovered as she went into remission (27).

### DNase1L3

The importance of normal clearance of cellular debris is demonstrated by another example of Mendelian inheritance in SLE. Linkage analysis of six consanguineous families with apparent autosomal recessive pSLE revealed a loss-of-function mutation in *DNASE1L3* (28). The children described in this study had very young age of onset and high disease activity with variable degree of renal involvement. Serologically, the patients all had hypocomplementemia, while most also had positive anti-dsDNA and antineutrophil cytoplasmic antibodies (28). Subsequently, DNASE1L3 mutations have been described in another family with childhood-onset SLE, as well as a family with three siblings affected by hypocomplementemic urticarial vasculitis (29, 30). This finding of monogenic SLE due to *DNASE1L3* deficiency followed previous observations of decreased DNase1 activity in adult SLE patients without Mendelian inheritance of disease (31). Heterozygous *DNASE1* mutations had also been described previously in SLE but definitive link to pathogenicity was still unclear (32).

DNase1 and DNase1L3 are related endonucleases that degrade extracellular DNA. Mice deficient in either DNase1 or DNase1L3 expression develop features similar to other mouse models of lupus (33, 34). Interestingly, the distinction between the two enzymes appears to be related to an additional C-terminal peptide on DNase1L3 that facilitates its ability to digest microparticle-bound DNA from apoptotic cells (35). Circulating microparticles from apoptotic cells in SLE patients are known to activate plasmacytoid and myeloid dendritic cells, resulting in the production of interferon α (IFN-α) (36). Overproduction of type I IFN is now understood to be a key feature of SLE [reviewed in detail by Eloranta and Ronnblom (37)]. Intracellular DNase1L3 may have other functions yet to be determined; for example, inhibition of DNase1L3 appears to inhibit inflammasome-mediated production of IL-1β (38).

### DNaseII

More recently, inherited deficiency of DNaseII has also been associated with an SLE-like phenotype. Rodero and colleagues described three children with loss-of-function mutations in *DNASE2*, resulting in neonatal onset of disease involving severe cytopenias, hepatosplenomegaly, and cholestatic hepatitis (39). All three later developed proteinuria with features of membranous glomerulonephritis; one child also developed deforming arthritis. In contrast to DNase1 and DNase1L3, DNaseII digests intracellular rather than extracellular DNA. Specifically, DNaseII recruitment to lysozymes is necessary for the cleavage of CpG DNA and the appropriate activation of TLR9 in response to infection (40). At the same time, DNaseII is important for the clearance of DNA from apoptotic cells within macrophage phagosomes; deficiency of this pathway leads to overproduction of IFN-β and TNF-α (41, 42).

### TREX1/DNaseIII

Appropriate clearance of cytosolic DNA is also necessary to prevent the development of autoimmunity. TREX1, also known as DNaseIII, is a 3′–5′ exonuclease that digests cytosolic DNA that would otherwise be immunostimulatory, inducing type I IFN production as part of antiviral immunity. The precise nucleic acid antigen that is responsible for triggering autoimmunity in the setting of TREX1 deficiency is not known but has been hypothesized to include endogenous retroelements as well as oxidized or otherwise damaged self DNA and RNA (43–47).

TREX1 has been linked to SLE due to the identification of two related disorders. Familial chilblain lupus (FCL) is an autosomal dominant condition characterized by vasculitic skin lesions and variable presence of autoantibodies (48). Aicardi–Goutieres syndrome (AGS) is another inherited disorder characterized by infantile neurological disease, hypergammaglobulinemia, chilblain lesions, and cerebrospinal fluid (CSF) lymphocytosis. Patients are noted to have high serum and CSF levels of IFN-α. Both FCL and AGS have been associated with defects in *TREX1*, among other genes (49, 50). The overlap between these conditions is further emphasized by the report of two siblings with homozygous *TREX1* mutations, one of whom has only chilblain lesions while the other has cerebral vasculitis reminiscent of AGS (51). Another report of a 4-year-old girl with classic features of SLE and central nervous system vasculitis was found by whole exome sequencing to have a homozygous mutation in *TREX1*, implying that TREX1 might play a broader role in the pathogenesis of non-Mendelian SLE (52). Further, heterozygous mutations in *TREX1* have been described at a higher rate in SLE patients as compared to healthy controls, and one particular *TREX1* haplotype has been associated with neurological manifestations in SLE (53, 54).

Taken together, inadequate clearance of extracellular, endosomal, and cytosolic DNA have all been associated with lupus-like autoimmunity. In these cases, self-DNA is inappropriately stimulates the activation of intracellular nucleic acid sensing pathways, resulting in the excessive production of type I IFN. Mutations in multiple other genes related to processing and sensing of intracellular nucleic acid have also been described to cause AGS and other monogenic autoimmune/autoinflammatory conditions, collectively termed “interferonopathies” (55). Notably, C1q deficiency is also characterized by excessive type I IFN, and clinical manifestations bear resemblance to other interferonopathies (56, 57). As overproduction of type I IFN is also a feature of non-Mendelian SLE, these monogenic disorders give insight into specific mechanisms by which IFN is induced in SLE, and how this influences the development of autoimmunity.

There may also be broader implications beyond lupus. In one cohort of 187 pediatric patients with a variety of autoimmune conditions without molecular genetic diagnosis, 69% had a positive IFN score (IS), as measured by overexpression of type I IFN-induced genes (58). As expected from prior studies, 82% of children with SLE and 75% of children with dermatomyositis had a positive IS. However, positive IS was also seen in conditions not typically characterized by type I IFN overproduction, including 29% of children with systemic juvenile idiopathic arthritis and 38% of children with non-interferonopathy autoinflammatory conditions (58). These findings raise the question of whether there may be subtypes of these conditions for which type I IFN has a pathophysiologic role, and whether these patients might be candidates for therapies that target IFN signaling.

### Protein Kinase C delta (PKCδ)

More recently, whole exome sequencing was used to identify mutation in *PRKCD* as the genetic defect underlying a family of siblings with early onset SLE and lupus nephritis (59). PKCδ, the serine/threonine kinase encoded by *PRKCD* is a component of multiple signal transduction cascades in different cell types. In B cells, PKCδ activation is downstream of signaling through both the B cell receptor and the BAFF receptor. PKCδ regulates BAFF-mediated survival and exerts a pro-apoptotic effect, promoting negative selection. Accordingly, deficiency of PKCδ leads to dysregulated B cell proliferation and loss of B cell tolerance (60). The described children with *PRKCD* mutation showed increased numbers of immature and transitional B cells with fewer switched and unswitched memory B cells (59). *In vitro*, B cells from these children demonstrated hyperproliferative response to stimulation and resistance to calcium flux-induced apoptosis (59). Because of these B cell abnormalities, rituximab was given with excellent response to two young siblings with SLE due to homozygous *PRKCD* mutation; these children had previously had disease that was refractory to other more standard treatments (61). Although *PRKCD* polymorphisms have not yet been studied at a population level in SLE, interestingly the heterozygous mother of these two siblings later developed SLE while pregnant with her third child (61). This finding raises the possibility that less severe defects in the PKCδ signaling pathway may have a broader role in the development of SLE in adults.

### Ras

There are multiple case reports of pSLE developing in patients with Noonan syndrome, an autosomal dominant disorder characterized by dysmorphic facial features, short stature, and cardiac and chest wall defects (62). Noonan syndrome and several related Noonan-like disorders are caused by mutations affecting genes in the Ras/MAPK signaling pathway. Examples of genes associated with these so-called “RASopathies” include *PTPN11, KRAS, NRAS, SOS1, SHOC2*, and *SHP2*, among others (63). The Ras/MAPK pathway is shared by multiple cellular processes, including proliferation, differentiation, and apoptosis. The coexistence of two relatively rare disorders within the same individual has raised questions about the role of Ras/MAPK signaling in SLE, as have two recent descriptions of children with SLE-like disease due to somatic gain-of-function (GOF) mutations in Ras pathway genes (64, 65). In the first case, a 4-year-old boy was diagnosed initially with Rosai-Dorfman disease with lymphadenopathy, hepatosplenomegaly, and pancytopenia. At age 7, he developed features of SLE with pericarditis, arthritis, and autoantibodies, and was eventually found to have somatic GOF mutation in *KRAS* (65). In the second case, a 3-year-old boy with chilblain lupus, pancytopenia, and autoantibodies was ultimately diagnosed with myelodysplastic syndrome due to somatic GOF mutation in *NRAS* (64). The contribution of Ras/MAPK signaling to SLE pathogenesis is further supported by a report that SHP2 activity is increased in one mouse model of lupus; the disease was ameliorated by treatment with a SHP2 inhibitor (66).

It remains unclear at this point how much continued characterization of monogenic lupus will contribute to our understanding of SLE physiology or treatment as a whole. The French GENetic and Immunologic Abnormalities in SLE (GENIAL/LUMUGENE) study is a longitudinal cohort describing the genetic and laboratory features of children with SLE. Initial findings were recently reported (67). The authors divide the cohort into three groups: (1) syndromic SLE, in which patients show clinical characteristics such as growth failure or intracranial calcifications suggestive of interferonopathies or other congenital disorder; (2) familial SLE, in which patients have either familial consanguinity or a first-degree relative with SLE; (3) all other early-onset SLE. Among the 64 patients described, 10 were considered syndromic, 12 familial, and 42 other. While the syndromic patients had younger age of onset than the other two groups, the authors were unable to find any other distinguishing physical or clinical characteristics, including response to therapy (67). More detailed immune profiling was not done in these patients, and as more targeted therapies become available, identification of specific pathway defects in familial cases may have more bearing on treatment.

## Molecular Profiling

Both pediatric and adult-onset SLE are characterized by clinical heterogeneity, presumably accompanied by pathophysiologic differences. A recent study used whole blood gene expression profiling from samples collected longitudinally to stratify pSLE patients into several groups (68). Expression data were categorized into distinct modules such as IFN response, plasmablast, neutrophil, erythropoiesis, and other gene signatures. The neutrophil, myeloid, and inflammation modules correlated with presence of lupus nephritis. Increased expression of the plasmablast module correlated with increased disease activity (68). Overall, differential expression of these modules was used to stratify pSLE patients into seven groups. As these stratifications did not necessarily correlate with distinct clinical features, the authors argue that molecular profiling, rather than clinical profiling, should be considered in the design of clinical trials for targeted therapies (68).

Immune cell and cytokine profiling using mass cytometry is another approach that has been used recently in pSLE. In a group of 10 clinically heterogeneous pSLE patients naïve to therapy, O’Gorman and colleagues found a shared signature of activated CD14^hi^ monocytes, characterized by increased monocyte chemoattractant protein (MCP-1), MIP1β, and IL-1RA production (69). Strikingly, the activated CD14^hi^ monocyte signature was seen in all 10 pSLE patients but none of the healthy controls, emphasizing the role of these cells as a common pathogenic factor in clinically variable SLE. The MCP-1/MIP1β/IL-1RA signature correlates strongly with disease activity and is at least partially dependent on type I IFN, although the authors did not find a type I IFN signature in all of the studied patients (69). Prior studies have suggested that IP-10, an IFN-γ-induced cytokine, may be a useful marker for disease activity. In a Chinese cohort of 46 pSLE patients, cytokine profiling revealed that IP-10 level performed better than anti-dsDNA, C3, or C4 in predicting active disease (70).

Autoantibody profiling has also been pursued in hopes of developing better biomarkers of disease activity. One study used an autoantigen array of over 140 antigens to study a cohort of new-onset pediatric SLE patients (71). The authors identified anti-BAFF antibodies in the majority of these patients and found that titer of these antibodies associated with disease activity level. The authors also identified autoantibodies associated with proliferative lupus nephritis. These include known antibodies such as anti-dsDNA and anti-C1q antibodies, but also antibodies against alpha-actinin, fibrinogen, collagens IV and X, aggrecan, and multiple histone proteins (71). While the anti-dsDNA and anti-C1q antibodies are known to correlate not just with nephritis but with flares of renal disease, the pathogenicity of these other antibodies is as yet undetermined.

## Conclusion

Pediatric SLE, while phenotypically often similar to adult-onset disease, may also present with more unusual or more severe features. In some cases, such as the neurologic disease associated with *TREX1* deficiency, it has been these differences that have highlighted the presence of an underlying pathogenic mechanism. The study of monogenic disease in children has opened new areas of investigation applicable to SLE as a whole, and it is very likely that more examples of this will be found in future. Molecular and immune profiling of pSLE patients has also generated insights into biomarker development and targets for therapy.

## Author Contributions

ML drafted the manuscript in its entirety.

## Conflict of Interest Statement

The author declares that the research was conducted in the absence of any commercial or financial relationships that could be construed as a potential conflict of interest.
